# Supplementary material for: A monoclonal antibody targeting a large surface of the receptor binding motif shows pan-neutralizing SARS-CoV-2 activity
Source: Nat Commun. 2024 Feb 5;15:1051. doi: 10.1038/s41467-024-45171-9 (PMC10844294; doi:10.1038/s41467-024-45171-9)
Supplement: Supplementary file 1 — Supplementary Information [file 41467_2024_45171_MOESM1_ESM.pdf]

A Monoclonal Antibody Targeting a Large Surface of the Receptor Binding Motif Shows Pan-Neutralizing SARS-CoV-2 Activity

Leire de Campos-Mata, Benjamin Trinité, Andrea Modrego, Sonia Tejedor Vaquero, Edwards Pradenas, Anna Pons-Grífols, Natalia Rodrigo Melero, Diego Carlero, Silvia Marfil, César Santiago, Dàlia Raïch-Regué, María Teresa Bueno-Carrasco, Ferran Tarrés-Freixas, Ferran Abancó, Victor Urrea, Nuria Izquierdo-Useros, Eva Riveira-Muñoz, Ester Ballana, Mónica Pérez, Júlia Vergara-Alert, Joaquim Segalés, Carlo Carolis*, Rocío Arranz*, Julià Blanco*, Giuliana Magri*

*Corresponding authors:

Carlo Carolis, carlo.carolis@crg.eu;

Rocío Arranz, rarranz@cnb.csic.es;

Julià Blanco, jblanco@irsicaixa.es;

Giuliana Magri, gmagri@imim.es

This file includes:

Supplementary Figures and legends 1-6
Supplementary Tables 1-5

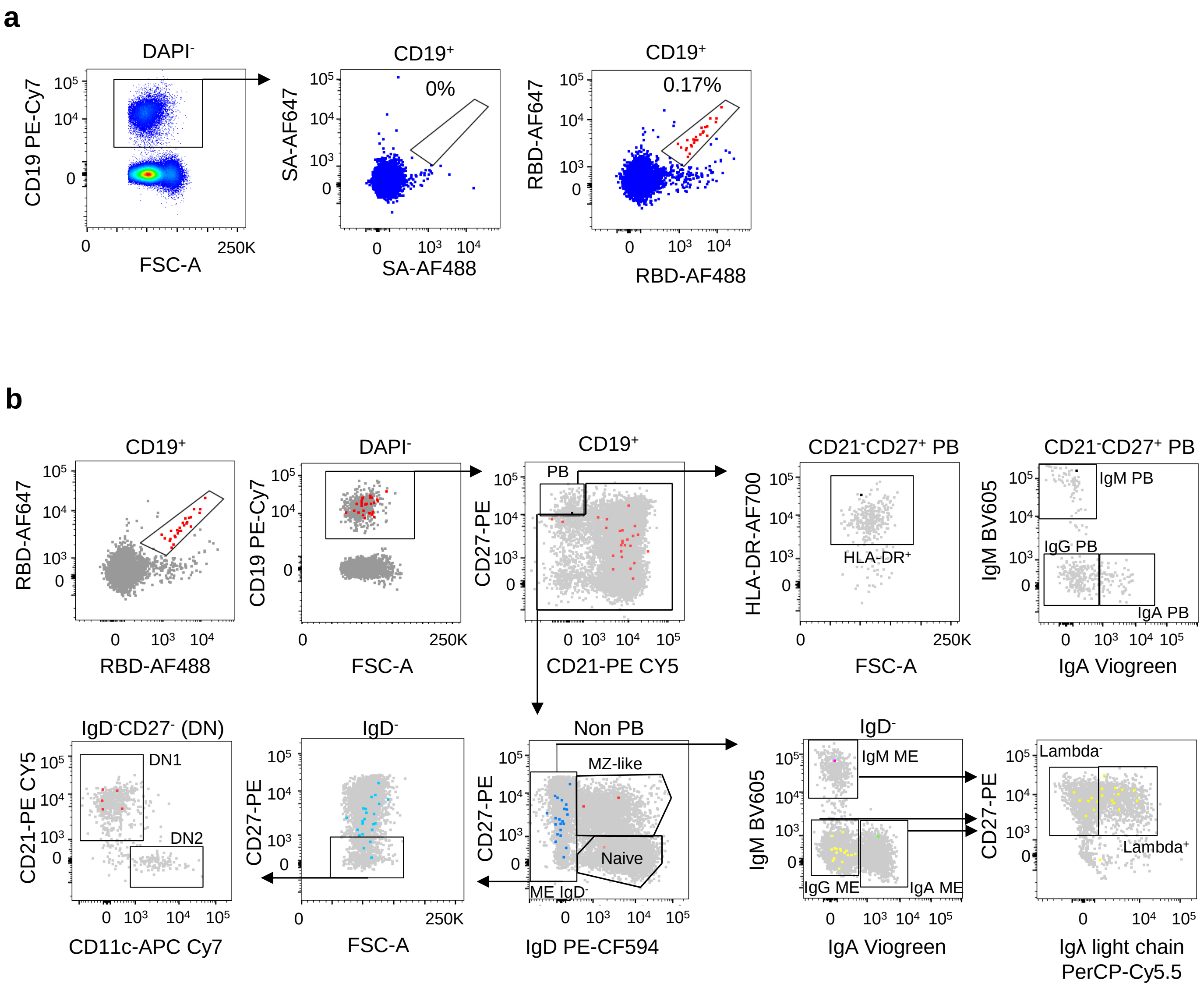

Supplementary Fig. 1. Gating strategy and phenotypic characterization of SARS CoV-2 WH1 RBD-specific B cells by flow cytometry. a Gating strategy used for single-cell index FACSorting of live DAPI^{-}CD19^{+}RBD^{+} B cells from a COVID-19 convalescent individual. Flow cytometry staining without (left) and with (right) two fluorescently labeled biotinylated RBD probes is shown. Numbers indicate the percentage of RBD-specific cells within total CD19^{+} B cells. Red large dots represent FACSorted cells that are positive for both RBD-AF647 and RBD-AF488. **b** Gating strategy used to define RBD-specific B cell populations: PB (CD19^{+}CD27^{++}CD21^{-}), IgM^{+} PB (PB CD21^{-}CD27^{+}IgM^{+}), IgG^{+} PB (PB CD21^{-}CD27^{+}IgM^{-}IgA^{-}), IgA^{+} PB (PB CD21^{-}CD27^{+}IgA^{+}), MZ-like (non-PB CD19^{+}CD27^{+}IgD^{+}), naïve (non-PB CD19^{+}CD27^{-}IgD^{+}), ME IgD^{-} (non-PB CD19^{+}IgD^{-}), IgM^{+} ME (non-PB CD19^{+}IgD^{-}IgM^{+}), IgA^{+} ME (non-PB CD19^{+}IgD^{-}IgA^{+}), IgG^{+} ME (non-PB CD19^{+}IgD^{-}IgM^{-}IgA^{-}), DN1 (non-PB CD19^{+}IgD^{-}CD27^{-}CD21^{+}CD11c^{-}), and DN2 (non-PB CD19^{+}IgD^{-}CD27^{-}CD21^{-}CD11c^{+}). PB, plasmablast; MZ, marginal zone; ME, memory; DN, double negative. RBD-specific cells are represented in colored large dots.

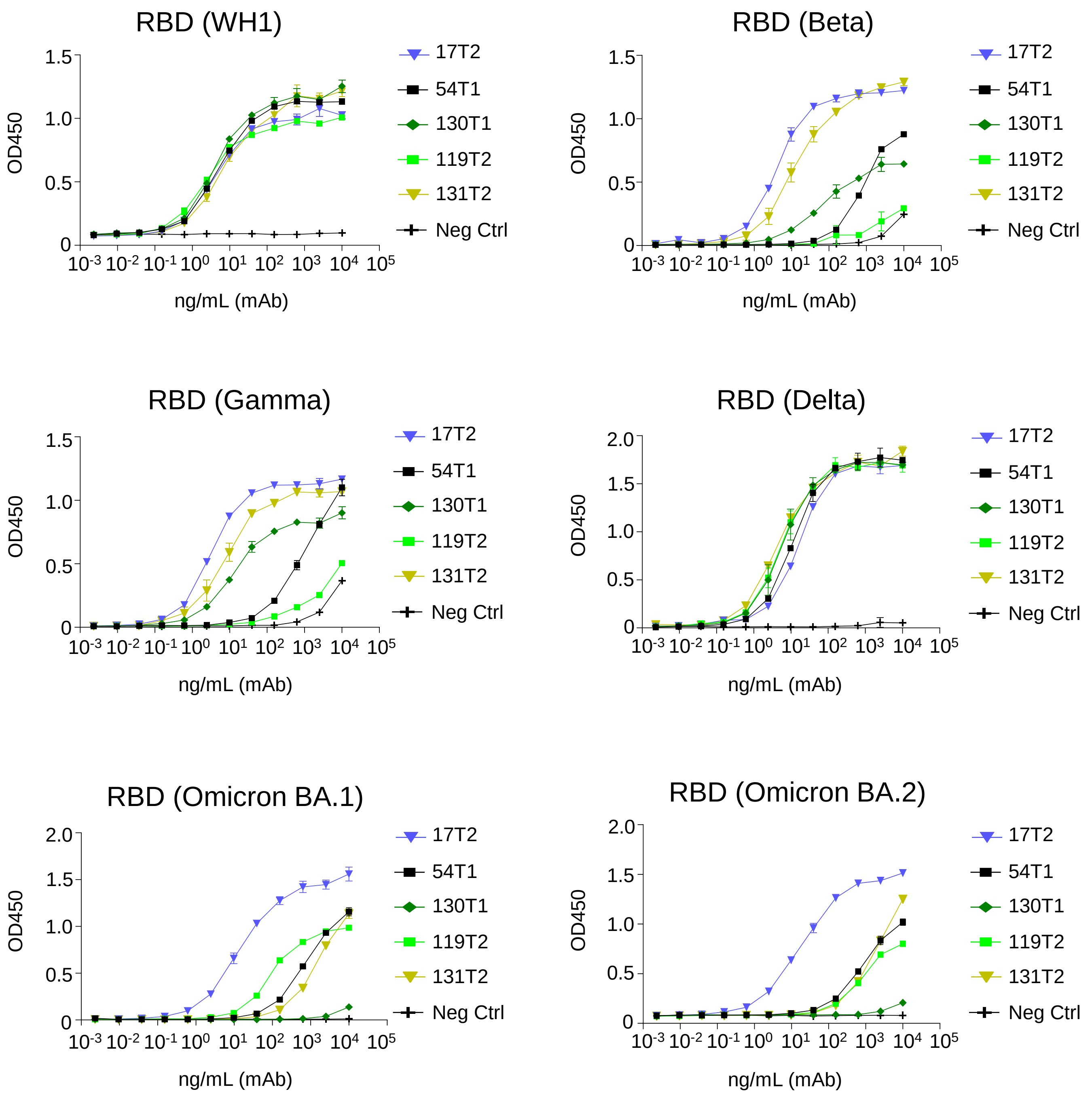

Supplementary Fig. 2. Reactivity of SARS-CoV-2 RBD-specific monoclonal antibodies. ELISA binding curves of serial dilutions of the mAbs to the spike RBD of SARS-CoV-2 from different variants, coated at equimolar concentrations. Graph bars represent the average ± SD of duplicate or triplicate samples. Human IgG1 purified from serum of a myeloma patient (Binding Site Company, BP078) was used as a negative control for binding. Source data are provided as a Source Data file.

a

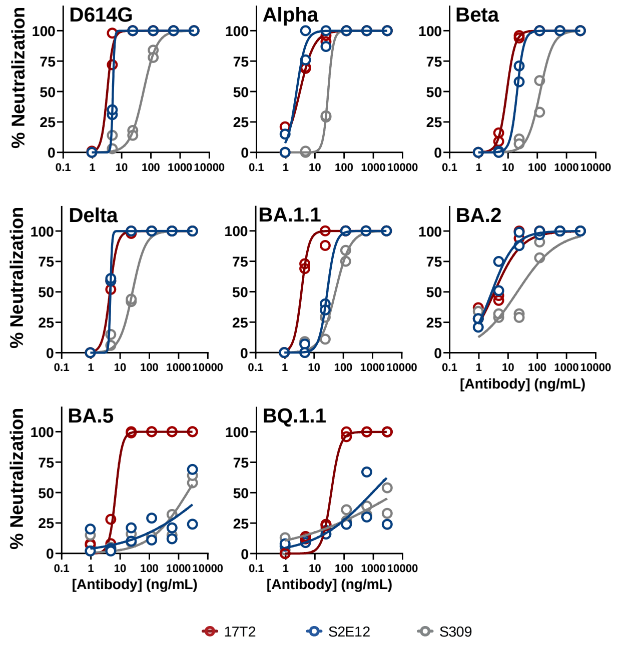

b

	17T2	S309	S2E12
D614G	3.2	56.0	5.0
Alpha	3.1	28.6	2.4
Beta	9.1	126.0	20.4
Delta	4.5	26.7	4.7
BA.1.1	3.7	53.7	28.3
BA.2	3.4	20.7	2.8
BA.5	7.1	>3,000.0	>3,000.0
BQ.1.1	36.2	>3,000.0	926.0

IC50 (ng/mL)

2.4 >3,000

c

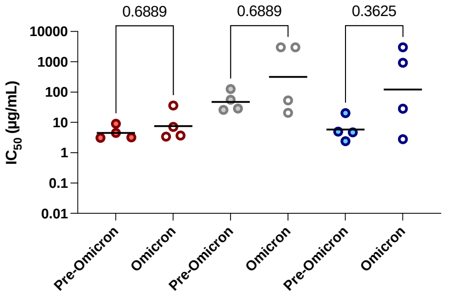

Supplementary Fig. 3. Broad neutralizing activity of 17T2 mAb. Authentic virus isolates were incubated with serial dilutions of the indicated mAbs and mixed with VeroE6 cells. IC_{50} values were determined based on protection against virus-induced cytopathic effect. **a** Neutralization curves of 17T2 (red), S309 (grey), and S2E12 (blue) mAbs against the indicated SARS-CoV-2 viral isolates. Duplicate values corresponding to a representative experiment are shown. **b** Heatmap showing IC_{50} values from panel **a** in ng/mL and color coded. Darker color corresponds to higher potency as indicated in the bottom of the Figure. **c** Impact of Omicron subvariants on virus neutralization capacity. IC_{50} values for pre-Omicron variants (D614G, Alpha, Beta and Delta) were grouped and compared to Omicron subvariants (BA.1.1, BA.2, BA.5, BQ.1.1). Solid bars show the geometrical mean. P values show individual corrected comparisons for each antibody using two-sided Kruskal-Wallis test with a global p value of 0.0359. Source data are provided as a Source Data file.

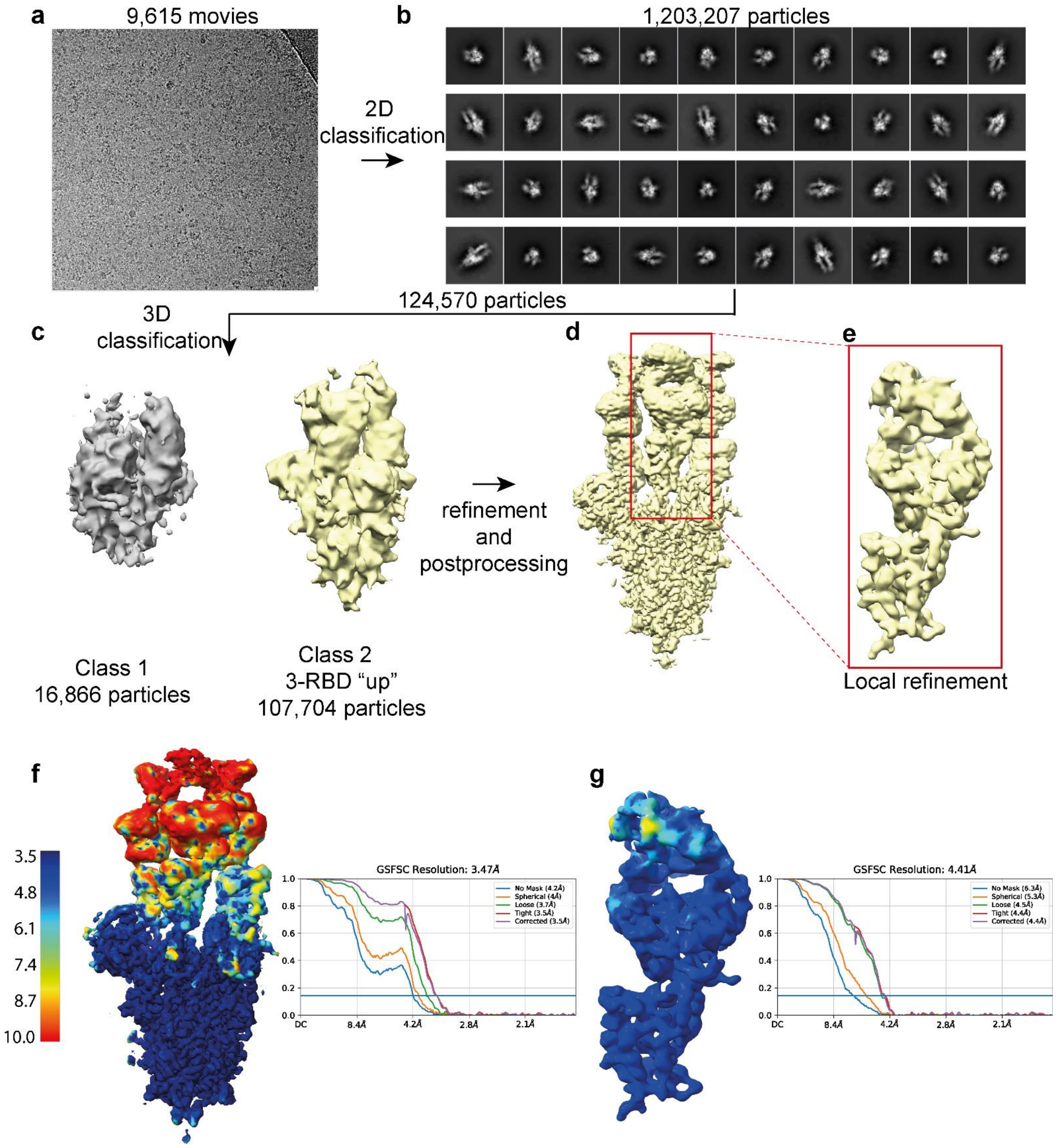

Supplementary Fig. 4. Cryo-EM data analysis for complex between the Omicron BA.1 spike trimer and the 17T2 Fab fragment. a Representative electron micrograph. **b** Representative 2D-class averages. **c** 3D classification. Left class contains low quality particles and right class contains high quality particles that were selected for refinement. **d** 3D reconstruction of complex with no symmetric imposed. **e** The boxed region contains one RBD complexed with one Fab in a refined map masked by local refinement. **f-g** Gold standard Fourier shell correlation (FSC) curve of final overall (left) and locally refined (right) maps and resolution

estimation based on 0.143 Fourier shell correlation criteria as indicated by a blue line. FSC curves are displayed after applying no mask (blue), a spherical mask (orange), a loose mask (green), a tight mask (red), or a tight mask with correction by noise substitution (purple) to both half maps before calculating FSC curves. The resolution at FSC = 0.143 is displayed for the corrected FSC curves (purple) as a blue line.

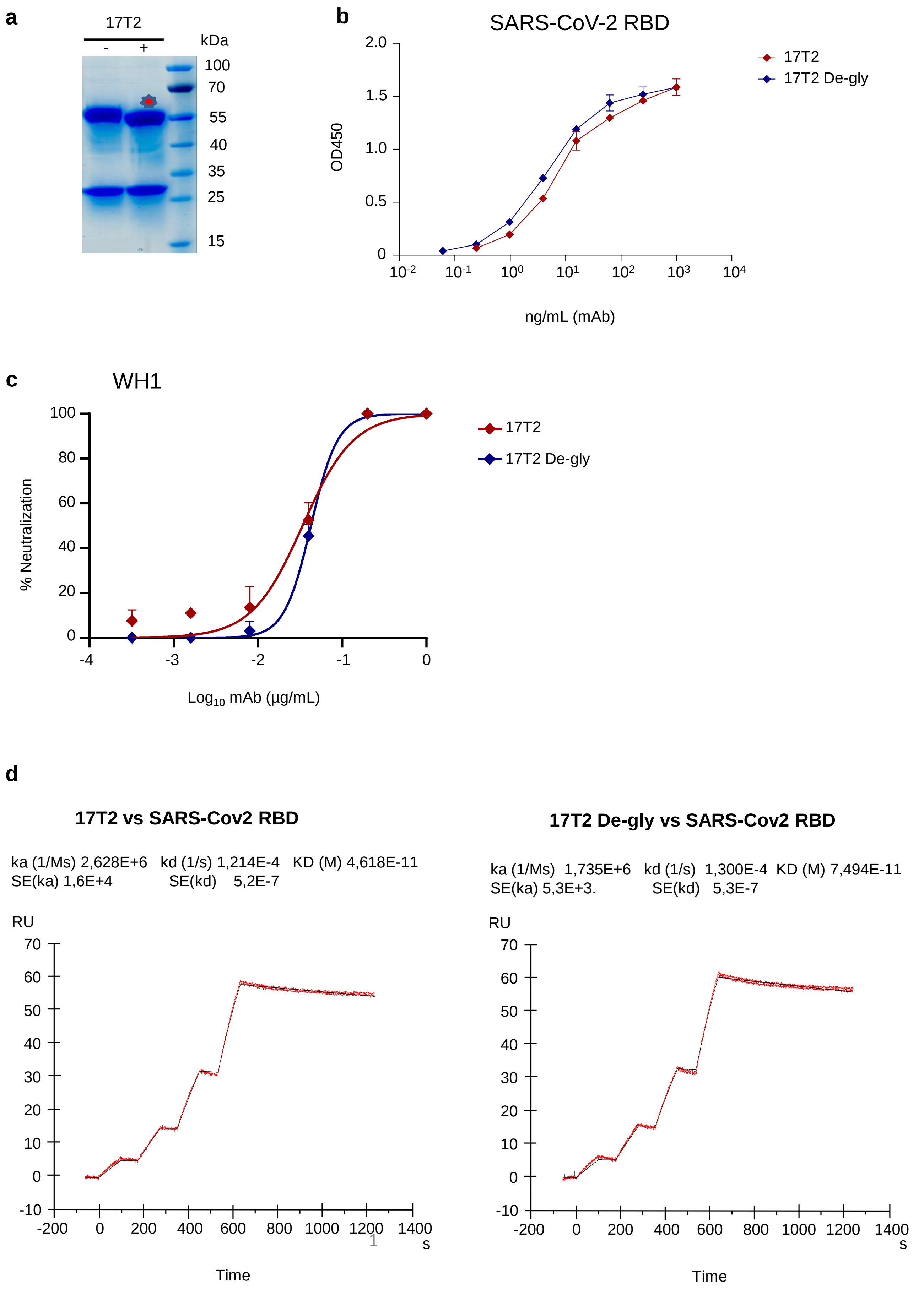

Supplementary Fig. 5. PNGase F treatment of purified 17T2 mAb. a Purified 17T2 mAb was incubated in the presence (+) or absence (–) of recombinant PNGase F and analyzed by SDS-PAGE. Red asterisks indicate the 17T2 de-glycosylated product (referred to as 17T2 De-gly). **b** ELISA binding curves of serial

dilutions of control treated 17T2 (red) and 17T2 De-gly (blue) mAbs to recombinant SARS-CoV-2 RBD, coated at equimolar concentrations. Graph bars represent the average ± SD of duplicate samples. **c** Neutralization curves of control treated 17T2 (red) and 17T2 De-gly (blue) mAbs against ancestral SARS-CoV-2 WH1 variant. Duplicate values corresponding to a representative experiment are shown. **d** The binding kinetics of no de-glycosylated form of 17T2 (left) and 17T2 De-gly (right) mAbs to recombinant SARS-CoV-2 RBD were obtained using the BIAcore T100 system in single-cycle mode. mAbs were captured on the chip, and serial dilutions of RBD (WH1) were then injected over the chip surface. The K_D is labelled accordingly. Source data are provided as a Source Data file.

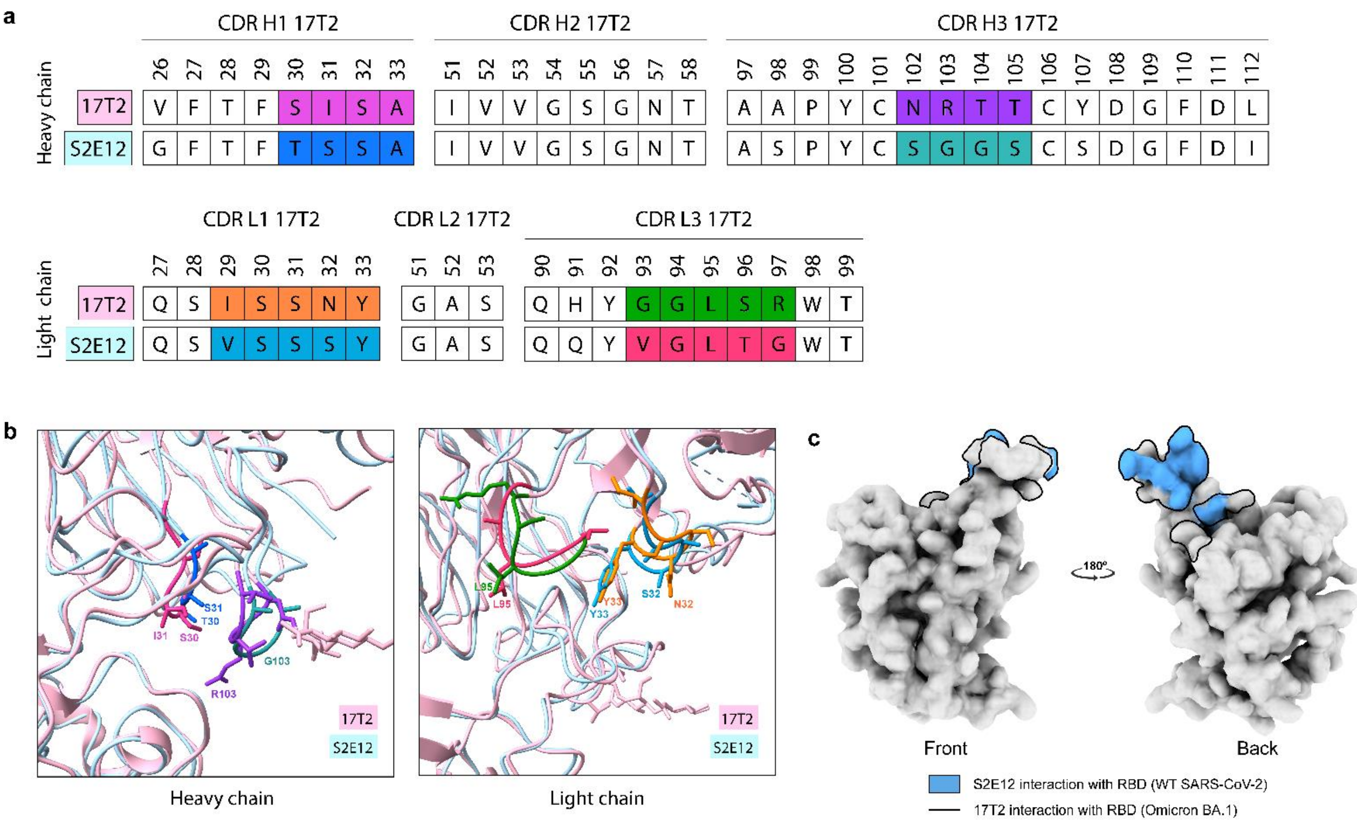

Supplementary Fig. 6. Comparative study of 17T2 Fab with similar antibody S2E12 Fab. a Comparison of the sequences of the CDRs of both the heavy and light chains between the 17T2 and S2E12 antibodies, showing differences between residues with colors. **b** Alignment of the RBD-Fab structures of 17T2 and S2E12 Fabs showing the side chains of the residues that are part of the regions with sequence differences (color codes as in **a**). **c** Different areas of interaction of 17T2 and S2E12 Fabs with the RBD of SARS-CoV-2 spike.

Supplementary Table 1. Phenotype and native isotype of the B cells originating the mAbs, and the antibody gene usage of the heavy and light chain variable regions.

Name	B cell of origin	
	B cell phenotype	Isotype
**17T2**	CD19^{+}HLA-DR^{+}CD27^{+}IgA^{+}CD21^{+}IgM^{-}IgD^{-}lambda^{-}	IgA
**54T1**	CD19^{+}HLA-DR^{+}CD27^{+}IgA^{+}CD21^{+}IgM^{-}IgD^{-}lambda^{-}	IgA
**130T1**	CD19^{+}HLA-DR^{+}CD27^{+}IgA^{-}CD21^{+}IgM^{-}IgD^{-}lambda^{-}	IgG
**119T2**	CD19^{+}HLA-DR^{+}CD27^{+}IgA^{-}CD21^{+}IgM^{-}IgD^{-}lambda^{+}	IgG
**131T2**	CD19^{+}HLA-DR^{+}CD27^{+}IgA^{-}CD21^{+}IgM^{-}IgD^{-}lambda^{-}	IgG

Name	IgHeavy			
	V call	D call	J call	% V identity
**17T2**	IGHV1-58*01	IGHD2-2*01,IGHD2-2*02,IGHD2-2*03	IGHJ3*01,IGHJ3*02	94,5
**54T1**	IGHV3-53*04	IGHD5-12*01	IGHJ6*02	96,6
**130T1**	IGHV1-46*01	IGHD5-18*01,IGHD5-5*01	IGHJ4*01,IGHJ4*02	96,6
**119T2**	IGHV3-9*01	IGHD2-15*01,IGHD2-21*01,IGHD2-21*02	IGHJ3*02	98
**131T2**	IGHV3-30*18,IGHV	IGHD5-18*01,IGHD5-5*01	IGHJ4*02	97,3

Name	IgLight			
	Chain	V call	J call	% V identity
**17T2**	**Kappa**	IGKV3-20*01	IGKJ1*01	96,5
**54T1**	**Kappa**	IGKV1-9*01	IGKJ1*01	98,6
**130T1**	**Kappa**	IGKV1-17*01	IGKJ1*01	98,6
**119T2**	**Lambda**	IGLV2-14*03	IGLJ3*02	98,3
**131T2**	**Kappa**	IGKV3-15*01	IGKJ4*01	97,6

Supplementary Table 2. Kinetic rate constants and affinities determined by surface plasmon resonance for the 17T2 mAb against SARS-CoV-2 RBD variants.

17T2			
**SARS-CoV-2 RBD variants**	**ka (1/Ms)**	**kd (1/s)[a]**	**K_D (nM)[b]**
WH1	2,62E+06	1,21E-4	0,0462
Alpha	9,61E+05	<1.0E-05	<0.1
Beta	2,30E+06	<1.0E-05	<0.1
Delta	3,32E+06	<1.0E-05	<0.1
Omicron BA.1	1,08E+07	<1.0E-05	<0.1
Omicron BA.2	5,90E+07	<1.0E-05	<0.1

a For most of the Biacore experiments, no decay in the binding signal was observed during the time allowed for dissociation. According to the ‘‘5% rule’’, the k_d can be resolved only if 5% or more of the bound material dissociates. Therefore, the upper limit on the K_d (in 1/s) is given by K_d < ln (0.95)/td, where td is the amount of time (in seconds) allowed for dissociation.

b K_D based on the kd limit determined by the ‘‘5% rule.’’

Supplementary Table 3. Pseudovirus spike mutations relative to ancestral WH1.

Name	Mutations
D614G	D614G
Alpha	H69-70del, Y144del, N501Y, A570D, D614G, P681H, T716I, S982A and D1118H
Beta	L18F, D80S, D215G, L242-244del, R246I, K417N, E484K, N501Y, D614G, A701V
Gamma	L18F, T20N, P26S, D138Y, R190S, K417T, E484K, N501Y, D614G, H655Y, T1027I, V1176F
Delta	T19R, 157-158 del, L452R, T478K, D614G, P681R, D950N
Mu	T95I, Y144S, Y145N, R346K, E484K, N501Y, D614G, P681H, D950N
BA.1	A67V, H69-70del, T95I, G142D, V143-145del, N211del, L212I, ins214EPE, G339D, S371L, S373P, S375F, K417N, N440K, G446S, S477N, T478K, E484A, Q493R, G496S, Q498R, N501Y, Y505H, T547K, D614G, H655Y, N679K, P681H, N764K, D796Y, N856K, Q954H, N969K, L981F
BA.2	T19I, L24S, P25-27del, G142D, V213G, G339D, S371F, S373P, S375F, T376A, D405N, R408S, K417N, N440K, S477N, T478K, E484A, Q493R, Q498R, N501Y, Y505H, D614G, H655Y, N679K, P681H, N764K, D796Y, Q954H, N969K
BA.4/5	T19I, L24S, P25-27del, H69-70del, G142D, V213G, G339D, S371F, S373P, S375F, T376A, D405N, R408S, K417N, N440K, L452R, S477N, T478K, E484A, F486V, Q498R, N501Y, Y505H, D614G, H655Y, N679K, P681H, N764K, D796Y, Q954H, N969K
BQ.1.1	T19I, L24S, P25-27del, H69-70del, G142D, V213G, G339D, R346T, S371F, S373P, S375F, T376A, D405N, R408S, K417N, N440K, K444T, L452R, N460K, S477N, T478K, E484A, F486V, Q498R, N501Y, Y505H, D614G, H655Y, N679K, P681H, N764K, D796Y, Q954H, N969K
XBB.1.5	T19I, L24S, del25-27, V83A, G142D, delY144, H146Q, Q183E, V213E, G252V, G339H, R346T, L368I, S371F, S373P, S375F, T376A, D405N, R408S, K417N, N440K, V445P, G446S, N460K, S477N, T478K, E484A, F486P, F490S, Q498R, N501Y, Y505H, D614G, H655Y, N679K, P681H, N764K, D796Y, Q954H, N969K
XBB.1.16	T19I, L24S, del25-27, V83A, G142D, delY144, H146Q, E180V, Q183E, V213E, G252V, G339H, R346T, L368I, S371F, S373P, S375F, T376A, D405N, R408S, K417N, N440K, V445P, G446S, N460K, S477N, T478R, E484A, F486P, F490S, Q498R, N501Y, Y505H, D614G, H655Y, N679K, P681H, N764K, D796Y, Q954H, N969K
EG.5.1	T19I, L24S, del25-27, Q52H, V83A, G142D, delY144, H146Q, Q183E, V213E, G252V, G339H, R346T, L368I, S371F, S373P, S375F, T376A, D405N, R408S, K417N, N440K, V445P, G446S, F456L, N460K, S477N, T478K, E484A, F486P, F490S, Q498R, N501Y, Y505H, D614G, H655Y, N679K, P681H, N764K, D796Y, Q954H, N969K
BA.2.86	ins16MPLF, T19I, R21T, L24S, del25-27, S50L, del69/70, V127F, G142D, delY144, F157S, R158G, N211I, delL212, V213G, L216F, H245N, A264D, I332V, G339H, K356T, S371F, S373P, S375F, T376A, R403K, D405N, R408S, K417N, N440K, V445H, G446S, N450D, L452W, N460K, S477N, T478K, N481K, delV483, E484K, F486P, Q498R, N501Y, Y505H, E554K, A570V, D614G, P621S, H655Y, N679K, P681R, N764K, D796Y, S939F, Q954H, N969K, P1143L

Supplementary Table 4. Representation of reported SARS-CoV-2 RBD mutations in key variants.

		Receptor-binding-domain mutations																																
		332	339	346	356	368	371	373	375	376	403	405	408	417	440	444	445	446	450	452	456	460	477	478	481	483	484	486	490	493	496	498	501	505
WT Sars-Cov-2		I	G	R	K	L	S	S	S	T	R	D	R	K	N	K	V	G	N	L	F	N	S	T	N	V	E	F	F	Q	G	Q	N	Y
Alpha	B.1.1.7																																Y	
Beta	B.1.351													N													K						Y	
Gamma	P.1													T													K						Y	
Delta	B.1.617.2																			R				K										
Epsilon	B.1.427/9																			R														
Zeta	P.2																										K							
Eta	B.1.525																										K							
Theta	P.3																										K						Y	
Iota	B.1.526																										K							
Kappa	B.1.617.1																			R				K			Q							
Lambda	C.37																			Q									S					
Mu	B.1.621			K																							K						Y	
Omicron	BA.1		D				L	P	F					N	K			S					N	K			A			R	S	R	Y	H
	BA.2		D				F	P	F	A		N	S	N	K								N	K			A			R		R	Y	H
	BA.4/BA.5		D				F	P	F	A		N	S	N	K					R			N	K			A	V				R	Y	H
	BQ.1.1		D				F	P	F	A		N	S	N	K	T				R		K	N	K			A	V				R	Y	H
	XBB.1.5		H	T		I	F	P	F	A		N	S	N	K		P	S				K	N	K			A	P	S			R	Y	H
	XBB.1.16		H	T		I	F	P	F	A		N	S	N	K		P	S				K	N	R			A	P	S			R	Y	H
	EG.5.1		H	T		I	F	P	F	A		N	S	N	K		P	S			L	K	N	K			A	P	S			R	Y	H
	BA.2.86	V	H		T		F	P	F	A	K	N	S	N	K		H	S	D	W		K	N	K	K	-	K	P				R	Y	H

Color highlighted boxes show the presence of RBD mutations in each SARS-CoV-2 variant compared to SARS-CoV-2 WH1 (same color codes as in Figure 4f). Green boxes depict mutated residues outside the interaction zone between 17T2 and the RBD, whereas yellow boxes depict mutated residues within the interaction zone.

Supplementary Table 5. Cryo-EM data collection, refinement and validation statistics

	**SARS-CoV2 Spike/17T2 Fab** (EMD-16453)	**RBD/17T2 Fab** (EMD-16473) (PDB 8C89)
**Data collection and processing**		
Magnification	120,000	120,000
Voltage (kV)	200	200
Electron exposure (e–/Å^2)	32	32
Defocus range (μm)	-1 to -2.5	-1 to -2.5
Pixel size (Å)	0.855	0.855
Symmetry imposed	C1	C1
Initial particle images (no.)	124,570	124,570
Final particle images (no.)	107,704	107,704
Map resolution (Å) (FSC threshold 0.143)	3.46	4.41
Map resolution range (Å)	3.46-10	4.41-10
**Refinement**		
Initial model used (PDB code)	-	-
Model resolution (Å) (FSC threshold 0.143)	-	-
Model resolution range (Å)	-	-
Map sharpening *B* factor (Å^2)	-125,1	-221,6
Model composition		
Non-hydrogen atoms	-	5,106
Protein residues	-	652
Ligands	-	4
B factors (Å^2)		
Protein	-	243.00
Ligand	-	301.85
R.m.s. deviations		
Bond lengths (Å)	-	0.006
Bond angles (°)	-	1.208
Validation		
MolProbity score	-	2.47
Clashscore	-	25.75
Poor rotamers (%)	-	0.71
Ramachandran plot		
Favored (%)	-	89.32
Allowed (%)	-	10.37
Disallowed (%)	-	0.31
