# Supplementary material for: A monoclonal antibody targeting a large surface of the receptor binding motif shows pan-neutralizing SARS-CoV-2 activity
Source: Nat Commun. 2024 Feb 5;15:1051. doi: 10.1038/s41467-024-45171-9 (PMC10844294; doi:10.1038/s41467-024-45171-9)
Supplement: Supplementary file 3 — Reporting Summary [file 41467_2024_45171_MOESM3_ESM.pdf]

nature portfolio

Corresponding author(s): Giuliana Magri, Julià Blanco, Carlo Carolis, Rocío Arranz

Last updated by author(s): Dec 19, 2023

Reporting Summary

Nature Portfolio wishes to improve the reproducibility of the work that we publish. This form provides structure for consistency and transparency in reporting. For further information on Nature Portfolio policies, see our Editorial Policies and the Editorial Policy Checklist.

Statistics

For all statistical analyses, confirm that the following items are present in the figure legend, table legend, main text, or Methods section.

n/a	Confirmed	
☐	☒	The exact sample size (*n*) for each experimental group/condition, given as a discrete number and unit of measurement
☐	☒	A statement on whether measurements were taken from distinct samples or whether the same sample was measured repeatedly
☐	☒	The statistical test(s) used AND whether they are one- or two-sided<br>*Only common tests should be described solely by name; describe more complex techniques in the Methods section.*
☐	☒	A description of all covariates tested
☐	☒	A description of any assumptions or corrections, such as tests of normality and adjustment for multiple comparisons
☐	☒	A full description of the statistical parameters including central tendency (e.g. means) or other basic estimates (e.g. regression coefficient) AND variation (e.g. standard deviation) or associated estimates of uncertainty (e.g. confidence intervals)
☐	☒	For null hypothesis testing, the test statistic (e.g. *F*, *t*, *r*) with confidence intervals, effect sizes, degrees of freedom and *P* value noted<br>*Give P values as exact values whenever suitable.*
☒	☐	For Bayesian analysis, information on the choice of priors and Markov chain Monte Carlo settings
☒	☐	For hierarchical and complex designs, identification of the appropriate level for tests and full reporting of outcomes
☒	☐	Estimates of effect sizes (e.g. Cohen's *d*, Pearson's *r*), indicating how they were calculated

Our web collection on statistics for biologists contains articles on many of the points above.

Software and code

Policy information about availability of computer code

Data collection	FACSDiva software (Becton Dickinson) was used for acquisition and sorting of RBD-specific B cells.
Data analysis	Prism v9.0 and R v4.1.1. was used to perform data analysis. Flow cytometry data were analyzed using FlowJo v10 software and FACSDiva software (Becton Dickinson). All cryo-EM image processing steps were performed with Scipion. Iterative manual model building and real-space refinement were carried out in Coot, Phenix and CCP-EM. The validation of the model was done with Molprobity sofware integrated in Phenix suite. UCSF Chimera and ChimeraX were used for map fitting and manipulation. PDBePISA and PDBsum servers were used for interaction analysis (residues involved and area of interaction) between 17T2 FAb and RBD.

For manuscripts utilizing custom algorithms or software that are central to the research but not yet described in published literature, software must be made available to editors and reviewers. We strongly encourage code deposition in a community repository (e.g. GitHub). See the Nature Portfolio guidelines for submitting code & software for further information.

Data

Policy information about availability of data

All manuscripts must include a data availability statement. This statement should provide the following information, where applicable:

- Accession codes, unique identifiers, or web links for publicly available datasets
- A description of any restrictions on data availability
- For clinical datasets or third party data, please ensure that the statement adheres to our policy

The data that support this study are available from the corresponding authors upon reasonable request. The cryo-EM data generated in this study have been deposited in the Electron Microscopy Data Bank under accession codes EMD-16453 for SARS-CoV-2 spike trimer in complex with three 17T2 Fabs and EMD-1643 for RBD/17T2 Fab. The associated atomic models generated in this study have been deposited in the Protein Data Bank under accession code 8C89. Source data are provided with this paper. Further information and requests for resources and reagents should be directed to and will be fulfilled by the lead Contact, Giuliana Magri (gmagri@imim.es).

Research involving human participants, their data, or biological material

Policy information about studies with human participants or human data. See also policy information about sex, gender (identity/presentation), and sexual orientation and race, ethnicity and racism.

Reporting on sex and gender	Sex and/or gender was not taken into consideration since the study derived from samples collected from a single individual
Reporting on race, ethnicity, or other socially relevant groupings	No socially constructed or socially relevant variables were used in this study
Population characteristics	The single individual involved in the study is 50 year old female caucasian. The individual had no prior history of disease/ treatment. Infection with COVID-19 presented mild symptoms.
Recruitment	Inclusion criteria for the study included the confirmation of diagnosis of SARS-CoV-2 infection by reverse transcription–quantitative polymerase chain reaction (RT-qPCR) of nasopharyngeal swab
Ethics oversight	All procedures were approved by the Ethical Committee for Clinical Investigation of the Institut Hospital del Mar d'Investigacions Mèdiques (Number 2020/9189/I)

Note that full information on the approval of the study protocol must also be provided in the manuscript.

Field-specific reporting

Please select the one below that is the best fit for your research. If you are not sure, read the appropriate sections before making your selection.

☒ Life sciences ☐ Behavioural & social sciences ☐ Ecological, evolutionary & environmental sciences

For a reference copy of the document with all sections, see nature.com/documents/nr-reporting-summary-flat.pdf

Life sciences study design

All studies must disclose on these points even when the disclosure is negative.

Sample size	Sample sizes were chosen based on prior experience and past power calculations. All experiments with statistical analysis were repeated at least two independent times, each with multiple technical replicates. Experimental size of animal cohorts was determined to fulfill 3R animal guiding principles while retaining statistical power based on prior experience performing studies in mice (see references 33, 52,and 53 in the manuscript).
Data exclusions	No data was excluded
Replication	All experiments had multiple biological and/or technical replicates as indicated in the figure legends
Randomization	For animal studies, mice were randomly assigned to treatment groups in an age-matched distribution. Randomization was no considered relevant for the experimental design of biochemical analyses in this study.
Blinding	Blinding was applied during all tissue analyses (RT-PCR, virus titration, Histology and IHC) but not during animal handling, supervision, sacrifice and tissue collection: to minimize cross-infection and or cross-contamination, all animal procedures were performed in the same order, starting with uninfected animals, followed by 17T2-treated animals, Isotype control-treated animal and finally untreated control infected animals. Animal work and tissue analyzes were performed by different teams (as described in the author contribution section).

Reporting for specific materials, systems and methods

We require information from authors about some types of materials, experimental systems and methods used in many studies. Here, indicate whether each material, system or method listed is relevant to your study. If you are not sure if a list item applies to your research, read the appropriate section before selecting a response.

Materials & experimental systems

n/a	Involved in the study
☐	☒ Antibodies
☐	☒ Eukaryotic cell lines
☒	☐ Palaeontology and archaeology
☐	☒ Animals and other organisms
☒	☐ Clinical data
☒	☐ Dual use research of concern
☒	☐ Plants

Methods

n/a	Involved in the study
☒	☐ ChIP-seq
☐	☒ Flow cytometry
☒	☐ MRI-based neuroimaging

Antibodies

Antibodies used

anti-human CD19 PE-Cy7 (clone: HIB19, source: Biolegend, Reference: Cat# 302216; RRID:AB_314246),
anti-human IgM Brilliant Violet 605 (clone: MHM-88, source: Biolegend, Reference: Cat# 314524; RRID:AB_2562374),
anti-human IgD PE-CF594 (clone: IA6-2, source: BD Biosciences, Reference: Cat# 562540; RRID:AB_11153129),
anti-human HLA-DR Alexa Fluor 700 (clone: L243, source: Biolegend, Reference: Cat# 307626; RRID:AB_493771),
anti-human CD11c APC-cy7 (clone: Bu15, source: Biolegend, Reference: Cat# 337217; RRID:AB_10661724),
anti-human CD27 PE (clone: O323, source: eBioscience, Reference: Cat# 12-0279-42; RRID:AB_10718394),
anti-human CD21 PE-cy5 (clone: B-ly4, source: BD Biosciences, Reference: Cat# 551064; RRID:AB_394028),
anti-human IgA VioGreen (clone: IS11-8E10, source: Miltenyi Biotec, Reference: Cat# 130-114-007; RRID:AB_2734098),
anti-human Ig light chain λ PerCP-cy5.5 (clone: MHL-38, source: Biolegend, Reference: Cat# 316617; RRID:AB_2561511),
horseradish peroxidase (HRP)-conjugated anti-human IgG secondary antibody (source: Southern Biotech, Reference: 2042-05, dilution 1:4000),
Human IgG1 purified from serum of a myeloma patient (source: Binding Site Company, Reference: BP078),
rabbit monoclonal antibody (source: Sino Biological, Reference: 40143-R019, dilution 1:15000),
17T2 mAb (reported in this study), 131T2 (reported in this study), 54T1 (reported in this study), 130T1 (reported in this study), 119T2 (reported in this study),
Human Anti-SARS-CoV-2 Spike-RBD S309 Neutralizing mAb (source: Cell Sciences, Reference: CPC525A),
Anti SARS-CoV-2 RBD Neutralizing antibody S2E12 (source: Proteogenix, Reference: PTXCOV-A579).
Recombinant anti-Human Immunodeficiency Virus type I gp120 monoclonal IgG1 antibody (source: Polymun Sientific, Reference: AB011).

Validation

All commercial antibodies were validated by the manufacturer per their associated DataSheets.
Company statement on validation: Each lot product is validated by QC testing with a series of titration dilutions.
The antibodies reported in this study were validated using SARS-CoV-2 RBD proteins by ELISA and its specificity was compared to a human IgG1 purified from serum of a myeloma patient (Binding Site Company, BP078; used as a negative control)

Eukaryotic cell lines

Policy information about cell lines and Sex and Gender in Research

Cell line source(s)	Expi293F human cells (Thermo Fisher Scientific), HEK293T cells overexpressing wild-type human ACE-2 (Integral Molecular), Vero E6 cells (ATCC CRL-1586)
Authentication	The cell lines were not authenticated
Mycoplasma contamination	All cell lines are routinely tested each month and were negative for mycoplasma
Commonly misidentified lines (See ICLAC register)	This study did not involve any commonly misidentified cell lines

Animals and other research organisms

Policy information about studies involving animals; ARRIVE guidelines recommended for reporting animal research, and Sex and Gender in Research

Laboratory animals

B6. CgTg(K18-ACE2)2Prlmn/J (or K18-hACE2) hemzygous transgenic mice (034860, Jackson Immunoresearch, West Grove, PA, United States) were bred, genotyped and maintained at CMCiB. A total of 44 adult K18-hACE2 hemizygous mice (aged 6-14 weeks) were used in this experiment. Specific housing conditions are described in the manuscript.

Wild animals	No wild animals were used in this study
Reporting on sex	K18-hACE2 mice were distributed in sex-balanced groups. Disaggregated data is available in the Source Data file.
Field-collected samples	No field-collected samples were used in this study
Ethics oversight	All animal procedures were performed under the approval of the Committee on the Ethics of Animal Experimentation of the IGTP and the authorization of Generalitat de Catalunya (code: 11222).

Note that full information on the approval of the study protocol must also be provided in the manuscript.

Plants

Seed stocks	Not applicable in this study
Novel plant genotypes	Not applicable in this study
Authentication	Not applicable in this study

Flow Cytometry

Plots

Confirm that:

☒ The axis labels state the marker and fluorochrome used (e.g. CD4-FITC).

☒ The axis scales are clearly visible. Include numbers along axes only for bottom left plot of group (a 'group' is an analysis of identical markers).

☒ All plots are contour plots with outliers or pseudocolor plots.

☒ A numerical value for number of cells or percentage (with statistics) is provided.

Methodology

Sample preparation	For the isolation of WH1 SARS-CoV-2 spike-specific B cells, 26.4 pmol His-Tagged Biotinylated SARS-CoV-2 (2019-nCoV) spike RBD Recombinant Protein (Sino Biological Inc.; cat number: 40592-VO8H-B) was firstly incubated for 1 hour with 3.78 pmol Streptavidin Alexa Fluor 647 (Thermo Fisher Scientific) and Streptavidin Alexa Fluor 488 (Thermo Fisher Scientific), separately. Next, PBMCs were incubated with the fluorescently labelled RBD probes and with a cocktail of fluorescent conjugated antibodies containing anti-CD19 Pe-Cy7, anti-IgM BV605, anti HLA-DR AF700, anti CD38 APC-cy7, anti Igλ light chain PerCPCy5.5 (all from Biolegend), anti-IgD PE CF594 (BD Bioscience), anti-CD21 PE-cy5 (BD Bioscience) and anti-IgA Viogreen (Miltenyi). Dead cells were excluded through the use of 4'-6'-diamidine-2'-phenylindole (DAPI) (Sigma).
Instrument	FACSAria II (BD Biosciences)
Software	FACSDiva software (Becton Dickinson) was used for acquisition and FlowJo for post-sorting analysis
Cell population abundance	WH1 SARS-CoV-2 RBD-specific B cells were approximately 0.15% within all live B cells (DAPI- CD19+)
Gating strategy	Alive DAPI- CD19+ RBD+ B cells were single-cell index sorted into empty 96-well PCR plates (VWR)

☒ Tick this box to confirm that a figure exemplifying the gating strategy is provided in the Supplementary Information.
